# Calreticulin Deficiency Disturbs Ribosome Biogenesis and Results in Retardation in Embryonic Kidney Development

**DOI:** 10.3390/ijms22115858

**Published:** 2021-05-30

**Authors:** Nazli Serin, Gry H. Dihazi, Asima Tayyeb, Christof Lenz, Gerhard A. Müller, Michael Zeisberg, Hassan Dihazi

**Affiliations:** 1 Clinic for Nephrology and Rheumatology, University Medical Center Göttingen, Robert-Koch-Strasse 40, 37075 Göttingen, Germany; nazli.serin@med.uni-goettingen.de (N.S.); gmueller@med.uni-goettingen.de (G.A.M.); michael.zeisberg@med.uni-goettingen.de (M.Z.); 2 Department of Hematology and Oncology, University of Medical Center Göttingen, Robert-Koch-Strasse 40, 37075 Göttingen, Germany; 3 Institute of Clinical Chemistry/UMG-Laboratories, University Medical Center Göttingen, Robert-Koch-Strasse 40, 37075 Göttingen, Germany; gryhelene.dihazi@med.uni-goettingen.de (G.H.D.); christof.lenz@med.uni-goettingen.de (C.L.); 4 School of Biological Sciences, University of the Punjab, Lahore 54590, Pakistan; asima.sbs@pu.edu.pk; 5 Bioanalytical Mass Spectrometry, Max Planck Institute for Biophysical Chemistry, 37077 Göttingen, Germany; 6 Center for Biostructural Imaging of Neurodegeneration (BIN), University Medical Center Göttingen, 37075 Göttingen, Germany

**Keywords:** calreticulin deficiency, nephrogenesis, ribosomal biogenesis

## Abstract

Nephrogenesis is driven by complex signaling pathways that control cell growth and differentiation. The endoplasmic reticulum chaperone calreticulin (Calr) is well known for its function in calcium storage and in the folding of glycoproteins. Its role in kidney development is still not understood. We provide evidence for a pivotal role of Calr in nephrogenesis in this investigation. We show that Calr deficiency results in the disrupted formation of an intact nephrogenic zone and in retardation of nephrogenesis, as evidenced by the disturbance in the formation of comma-shaped and s-shaped bodies. Using proteomics and transcriptomics approaches, we demonstrated that in addition to an alteration in Wnt-signaling key proteins, embryonic kidneys from Calr^−/−^ showed an overall impairment in expression of ribosomal proteins which reveals disturbances in protein synthesis and nephrogenesis. CRISPR/cas9 mediated knockout confirmed that Calr deficiency is associated with a deficiency of several ribosomal proteins and key proteins in ribosome biogenesis. Our data highlights a direct link between Calr expression and the ribosome biogenesis.

## 1. Introduction

Kidney organogenesis is characterized by a succession of morphogenetic events that is driven by cell growth and differentiation. During nephrogenesis, the mesenchymal–epithelial transition (MET) and the ureteric bud (UB) branching, which is the result from reciprocal induction between the UB and the metanephric mesenchyme (MM) [1], are the driving forces for the nephron formation. During this process a complex and reciprocal interaction of various signaling pathways is necessary to orchestrate epithelial differentiation and the formation of the nephron [1,2,3]. Among the key pathways in this process, the glia derived neurotrophic factor (Gdnf) signaling via its Ret receptor plays a central role during a process called ureteric budding. Disruption of this signaling may cause ectopic ureter or renal agenesis when the signaling is completely absent [4]. Apart from the Gdnf/Ret signaling, the canonical Wnt/β-catenin signaling is known to coordinate multiple aspects of renal development within both the MM and UB [5] and the inhibition of canonical Wnt signaling in the ureteric bud lineage and nephron progenitors results in renal agenesis [6]. Maintenance of the transcriptional reprogramming during nephrogenesis depends on chromatin accessibility [7]. We demonstrated that heterochromatin proteins, which are known to be involved in the epigenetic regulation of gene silencing, are indispensable for maintaining the balance between branching activators and inhibitors in the early stage of development [7].

Calreticulin (Calr) is a promiscuous chaperone of the endoplasmic reticulum (ER) with a high calcium-binding capacity and low affinity, which are crucial for Ca^2+^ sequestration to the ER and different cellular processes including signal transduction, gene expression, and protein trafficking [8,9]. The mechanisms addressing the role of Calr in diseases are mainly based on Ca^2+^ chelation by the Ca^2+^-binding C-terminal domain of Calr and its role in the unfolded protein response (UPR) [10,11]. UPR can play a cytoprotective role during a challenge with misfolded proteins or can be detrimental to the cells as sustained UPR signaling induced apoptosis. Although few studies addressed the potential role of Calr in diseases through regulation of transcription, ER chaperone function of Calr remains one of the crucial mechanisms for the cell’s fate [9,12]. Prolonged ER stress and protein misfolding are prominent in various renal diseases such as glomerulopathies, acute kidney injury, diabetic nephropathy, renal fibrosis, and chronic kidney disease [13,14].

The role of Calr in embryonic development is still not clear. Calr knockout causes embryonic lethality during the developmental stage E14.5 due to impaired cardiac development [15]. The consequences of Calr deficiency on the molecular mechanisms of the kidney embryonic development, however, are not yet fully understood. In the present study we performed comparative transcriptomic and proteomic analyses of the embryonic kidney from the three genotypes Calr^+/+^, Calr ^+/−^, and Calr^−/−^ and highlighted the impact of Calr knockout on nephrogenesis and on the expression of ribosomal proteins.

## 2. Results

### 2.1. Calreticulin Knockout Results in Morphological and Histological Abnormalities of the Kidney and in Impaired Renal Branching

**Figure 1:**
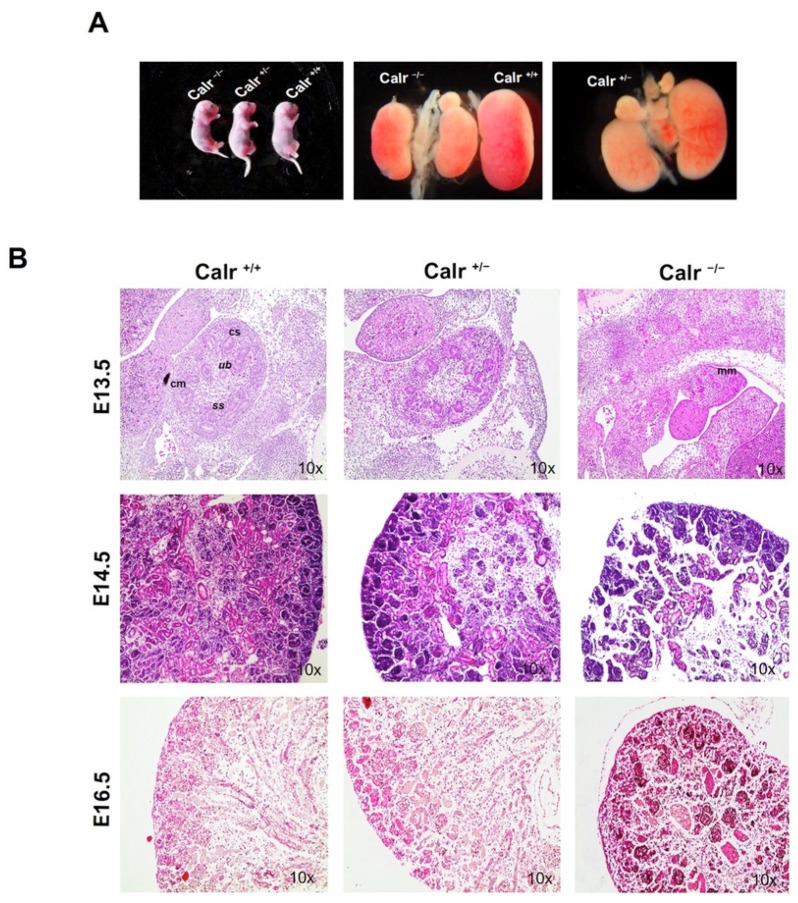
Calr^−/−^ embryos and embryonic kidney displaying abnormalities. (**A**): Mouse embryos from the three different genotypes showing abnormal Calr^−/−^ embryos. The comparison of the size and phenotype of embryonic kidneys from the three different genotypes revealed an alteration in nephrogenesis in Calr^−/−^ mouse embryos. (**B**): HE/PAS staining of embryonic kidney sections from different embryonic stages (E13.5, E14.5, and E16.5) and from the three genotypes (Calr^+/+^, Calr^+/−^, and Carl^−/−^) showing the metanephros, including the outgrowth of the ureteric bud (ub), comma-shaped (cs), s-shaped body (ss), cap mesenchyme (cm).

Genetic knockout of Calr in mice causes early embryonic lethality due to ventricular septal defects [15]. In order to investigate whether Calr knockout impacts kidney development, mouse embryos at stage E13.5 were prepared from Calr^+/−^ pregnant mice (Figure 1A). The Calr^−/−^ embryos showed significant growth alteration compared to Calr^+/+^ and Calr^+/−^ and revealed significant impairment in the embryonal development. Gross morphology of the embryos and the kidneys showed significant decreases in size between Calr^+/+^ and Calr^−/−^ mice with Calr^−/−^ mice showed the greatest abnormality (Figure 1A). To illustrate the impact of the Calr knockout on kidney development and structure, embryos at three different stages of development (E13.5, E14.5, E16.5) and from the three genotypes (Calr^+/+^, Calr^+/−^ and Calr^−/−^) were sacrificed and the kidneys were obtained. The tissue sections showed different developing stages of the kidney, which progresses through complicated morphological structures as evidenced by PAS and HE-staining of tissue sections. Kidneys from the same embryonic stage with different genotypes showed different levels of development (Figure 1B). Moreover, significant differences in kidney structures were observed especially when comparing Calr^−/−^ to Calr^+/+^ and Calr^+/−^. These differences also remain in advanced stages of nephrogenesis (Figure 1B). Calr^−/−^ kidneys shows severe impairment compared to Calr^+/+^ and Calr^+/−^ (Figure 1B). At stage E13.5 the size of the Calr^−/−^ kidney was smaller and the number of ureter buds and ureter bud branches were significantly lower. Similar observations were made at stages E14.5 and E16.5. Organ culture of the embryonic kidneys from the three embryo genotypes unveiled substantial disorder in UB branching and kidney growth. To visualize the renal structures, cultured rudiments were stained with laminin as a marker for the basal membrane and with *Dolichos biflorus* agglutinin (DBA) lectin to visualize the UB.

**Figure 2:**
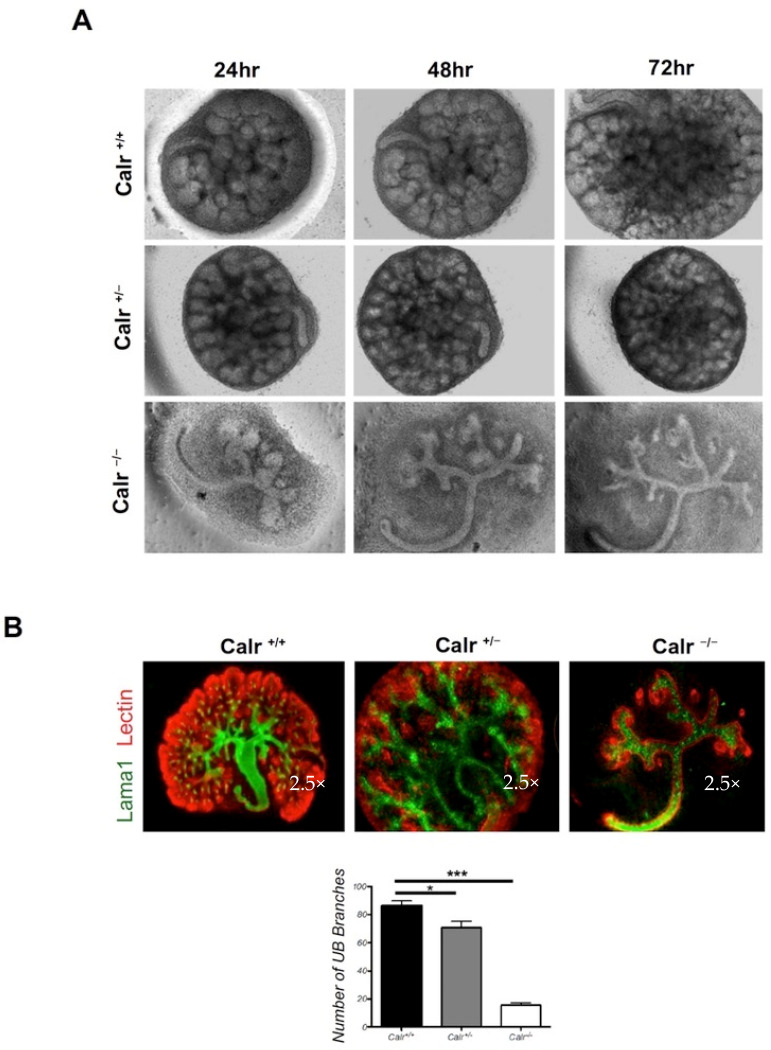
Calr^−/−^ mouse kidney rudiments display a severe alteration in growth and UB branching. (**A**): Kidney rudiments from Calr^+/+^ Calr^+/−^ and Calr^−/−^ (E13.5) were isolated and cultured ex vivo for three days. Calr^−/−^ kidney rudiments showed an overall alteration in growth and a significant alteration in ureteric bud branching. (**B**): Immunofluorescence staining of kidney rudiments after three days of culture. A co-staining of laminin (red) and DBA lectin (green) was performed to visualize the different structure of the rudiments. The branching quantification was achieved by counting the number of branches of the ureteric bud. The quantification is presented as a bar chart with error bars. Each bar represents the branch’s number means ± s.d. from six cultured rudiments. Significant differences: (*) *p* < 0.05, (***) *p* < 0.001.

The combined fluorescence staining of the cultured kidney rudiments showed a significant branching impairment accompanying Calr deficiency and resulting in an overall retardation in kidney development (Figure 2A,B). At the time of isolation, the Calr^−/−^ kidney rudiments showed 6–10 UB tips, while the Calr^+/+^ and Calr^+/−^ rudiments exhibited 20–30 UB tips. The Calr^+/+^ and Calr^+/−^ cultured rudiments developed normally during the culture period (72 h) and showed a well-branched UB (85 ± 9, *n* = 6 each) as well as different known structures from in vivo developing kidney. The cultured kidney demonstrated pre-tubular aggregates, renal vesicles, and cap mesenchyme (Figure 2A,B). In contrast, the Calr^−/−^ rudiments failed to develop normally and showed a significant alteration in the UB branching (15 ± 3 *n* = 6) (Figure 2B).

### 2.2. Calr Deficiency Is Associated with Large and Significant Transcriptome Alterations

**Figure 3:**
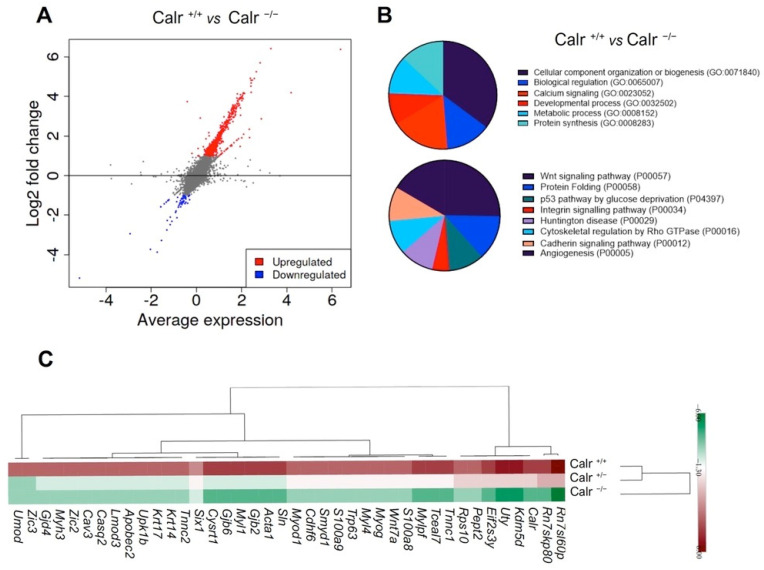
Comparative gene expression analysis of calreticulin knockout mice. (**A**): MA plot showing wide scale up-regulatedand down-regulated genes in Calr^+/+^ mice versus Calr^−/−^. Quantitative tests of the fold change (based on the normalized log transformed read count) were performed using Fisher’s exact test with a Benjamini–Hochberg correction (*p* < 0.05) and non significant genes are represented in gray. MA plot is used for displaying the differentially expressed genes in Calr^+/+^ vs. Calr^−/−^. (**B**): Distribution of the biological processes of the down-regulated genes in Calr^−/−^ compared to Calr^++-^. The classification of the identified genes was carried out using a DAVID bioinformatics tool. The gene symbol was used to categorize the gene ontology annotations, e.g., biological processes. (**C**): Enrichment analysis of the top genes found to be significantly regulated between the three genotypes (Calr^+/+^, Calr^+/−^ and Calr^−/−^). The comparative analysis is represented as a heatmap (FC < −2 and FC > 2).

The morphological and histological investigations showed an impairment in kidney development in Calr^−/−^ mouse embryos compared to Calr^+/+^ and Calr^+/−^. In order to investigate whether the Calr knockout impacted the expression of nephrogenesis key proteins and pathways, entire transcriptome analyses of the kidney rudiments were performed from the three embryo genotypes. In order to assess the differentially expressed genes between Calr^+/+^, Calr^+/−^, and Calr^−/−^ kidney samples, quantitative tests based on read counts were performed using Fisher’s exact test with a Benjamini–Hochberg adjustment for multiple tests. The Supplementary Figures S1 and S2 display the heat map of the comparative analysis between the gene expressions in the kidney rudiments from Calr^+/+^, Calr^+/−^, and Calr^−/−^. In order to obtain a comprehensive overview of the gene expression changes between Calr^+/+^ and Calr^−/−^ kidney, a MA-plot was created with the transformed fold change (FC) of expression between Calr^+/+^ and Calr ^−/−^ to log2 of transformed average expression level for each gene across all the samples (Figure 3A, Supplementary Figure S1). The MA-plot shows differentially regulated genes in Calr^+/+^ compared to Calr^−^^/−^. A functional classification of the regulated genes according to biological process revealed an alteration of the developmental process in Calr^−/−^ kidneys (Figure 3B, Supplementary Table S1). A closer investigation into the pathway annotation of the regulated genes revealed an aberration of two main processes: the Wnt signaling and the protein folding as the majority of the regulated proteins were found to be involved in these two main pathways (Figure 3B). Hierarchical clustering and k-means clustering on expression profiles confirmed that the embryonic kidney transcriptomes of Calr^+/+^ and Calr^+/−^ are very similar but differ significantly from the transcriptomes of the Calr^−/−^ (Figure 3C, Supplementary Figures S1 and S2).

**Figure 4:**
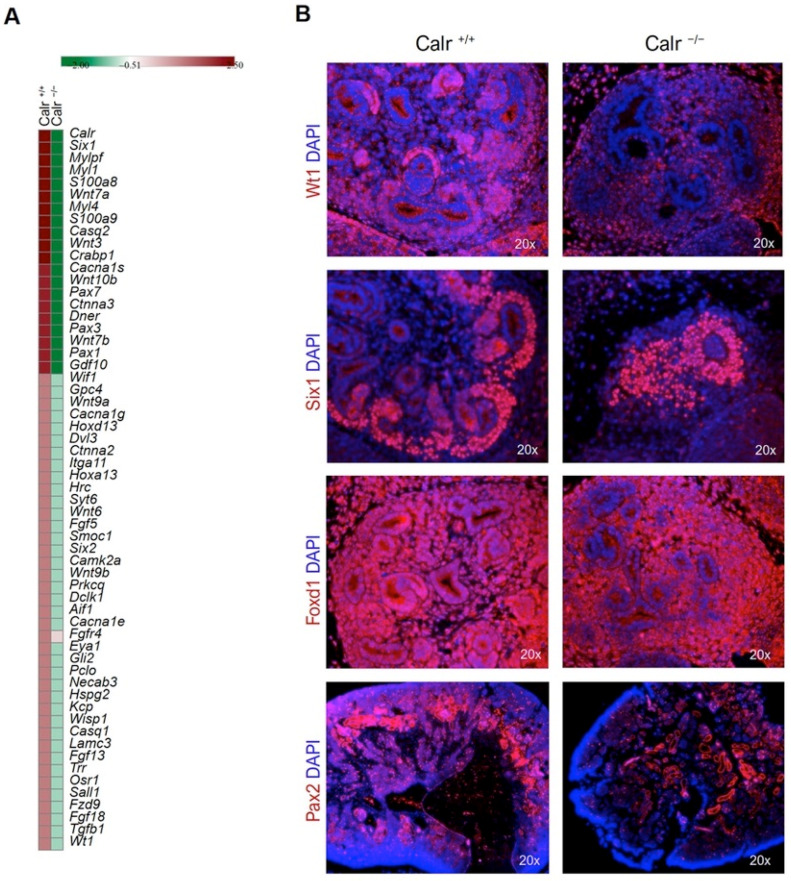
Immunofluorescent staining of sections from the embryonic kidneys. (**A**): Heat map of the essential genes and transcription factors for developmental processes found to be down-regulated in Calr^−/−^ compared to Calr^+/+^ (FDR < 0.1, FC > 2). (**B**): Embryonic kidneys with three genotypes (Calr^+/+^, Calr^+/−^ or Calr^−/−^) were harvested and prepared for immunofluorescence staining. Wt1, Six2, and Pax2 staining was carried out and the slides were subsequently counterstained with DAPI for nuclear visualization on a magnification of 20×.

Our transcriptome analysis data showed that the knockout of Calr was accompanied by an alteration in the expression of key proteins in kidney development (Figure 3C and Figure 4A, Supplementary Table S2) alongside the key proteins in Wnt signaling and a large number of nephrogenic genes were down-regulated or not detected in the Calr^−/−^ kidney rudiments (Figure 3C, Figure 4A). Among others, transcription factors, such as *Six1* and *Six2*, previously reported to function in different stages of nephrogenesis were significantly down-regulated in Calr^−/−^ embryonic kidney. *Osr1* is one of the genes whose expression is required for *Eya1, Pax2, Six2, Sall1*, and *GDNF* and the expression of this gene was almost absent in Calr^−/−^. In order to investigate the impact of the alteration of Wnt signaling and the nephrogenic key genes on kidney development in Calr knockout mice, immunofluorescence staining with markers of the embryonic development was carried out in kidney sections from embryos at stage E14.5. The staining clearly confirmed the alteration of the investigated genes in Calr^−/−^ (Figure 4B). Additionally, when compared to Calr^+/+^ and Calr^+/−^, the Calr^−/−^ kidney lacks a clear nephrogenic zone (Figure 4B) as evidenced by the staining and this confirms severe disturbance in kidney development in Calr^−/−^ embryos.

### 2.3. Comparative Proteomic Analyses Identified Significant Alterations in the Proteome of the Calr^−/−^ Embryonic Kidney

**Figure 5:**
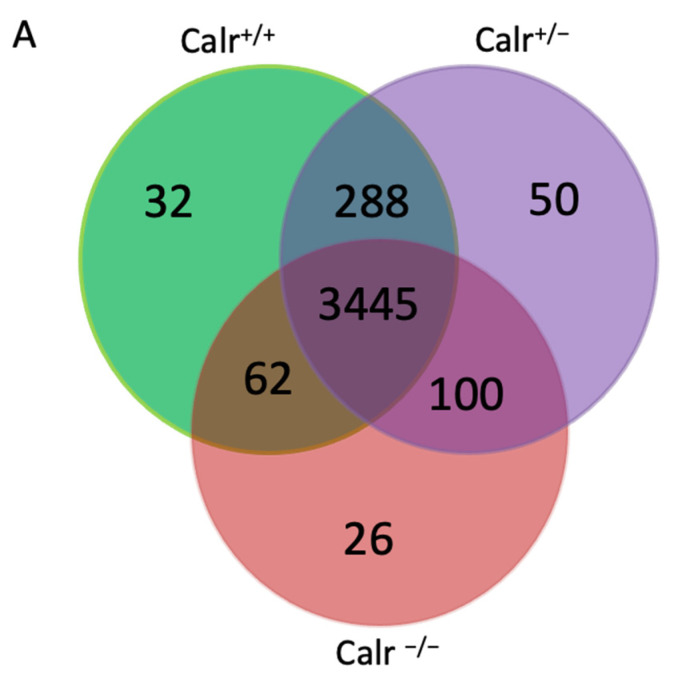

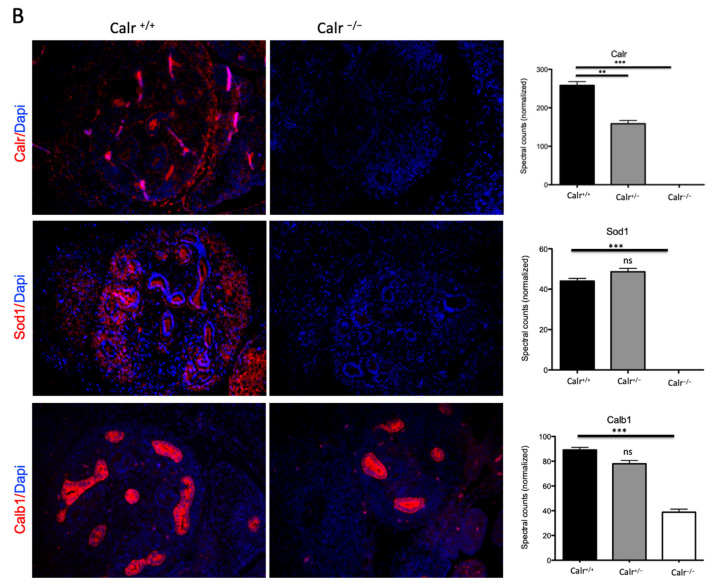
Investigation of the proteome alteration in Calr deficient embryonic kidney. The embryonic kidney proteome was analyzed by combining 1DE and mass spectrometry. (**A**): A Venn diagram illustrating the distribution of proteins identified in different kidney genotypes. The Venn diagram shows the proteins common in all three groups or in two groups and highlights proteins expressed in only one of the groups. (**B**): Immunofluorescence staining and mass spectrometric quantification of selected proteins down-regulated in Calr^−/−^ compared to Calr^+/+^ and Calr^+/−^. Significant differences were accorded to: (**) *p* < 0.01, (***) *p* < 0.001.

Morphological and histological analyses showed that a restriction of Calr is problematic for the embryonic development of the kidney. To further understand the role of Calr in kidney embryonic development, wide proteome analyses were carried out on embryonic kidney using two strategies. In the first experiments we used the 2D-gel electrophoresis to compare the embryonic kidney proteomes from Calr^+/+^ and Calr^+/−^ mouse embryos from stage E13.5. The 2D-pattern showed a significant alteration in the proteome of Calr^+/−^ kidneys (*p* < 0.05) (Supplementary Figure S3A). The identification of the differentially abundant proteins revealed an alteration in the expression of proteins involved in stress pathways and in RNA metabolism in Calr^+/–^ embryonic kidney (Supplementary Figure S3B,C). In order to better explore the proteome alteration in Calr^+/−^ and Calr^−/−^ compared to the Calr^+/+^ kidney, we performed mass spectrometry-based proteome profiling (Figure 5, Supplementary Tables S3–S8). An overlap analysis showed a high reproducibility of protein detection between genotypes (Figure 5A). Statistical analysis exhibited significant alterations in protein expression between Calr^+/+^ vs. Calr^−/−^ (Figure 5A, Supplementary Tables S3 and S4, Supplementary Figure S4A.), Calr^+/^^−^ vs. Calr^−^^/−^ (Figure 5A, Supplementary Tables S5 and S6, Supplementary Figure S4B), and Calr^+/+^ vs. Calr^+/^^−^ (Figure 5A, Supplementary Tables S7 and S8, Supplementary Figure S4C). Immunofluorescence staining confirmed the knockout of Calr in Calr^−^^/−^ kidneys. Additionally, we confirmed the proteomics findings for two down-regulated proteins in Calr^−^^/−^: Calbindin 1(Calb-1) and Superoxide dismutase 1 (Sod1). In the case of Sod1, both proteomic and transcriptomic data revealed a knockout of Sod1 in the Calr^−^^/−^ embryonic kidney and immunofluorescence staining confirmed the deficiency of Sod1 in the Calr^−^^/−^ embryonic kidney (Figure 5B). Sod1 is an antioxidant metalloenzymes that plays an important role in the detoxification of reactive oxygen species (ROS) by converting free superoxide radicals into hydrogen peroxide for further detoxification by cellular catalases, thus preventing toxicity. Calb-1 is the major intracellular calcium binding protein in distal convolute tubule (DCT) and plays a key role in the transcellular Ca^2+^ reabsorption. As an intracellular Ca^2+^-buffer and Ca^2+^-transit protein, Calb-1 transports Ca^2+^ from the apical to the basolateral side without significant modification in the intracellular Ca^2+^-concentration. The link between the Calr knockout and the alteration in the expression of both proteins is not clear and requires further investigation.

### 2.4. Gene Ontology Classification and Protein–Protein Interaction Network Analyses

The volcano plots and the pie chart analyses showed that Calr expression alteration results in global changes in the embryonic kidney proteome (Supplementary Figure S4). In order to gain more information on the biological mechanisms associated with the embryonic kidney development alteration in Calr^−/−^ mice, we combined DAVID bioinformatics with information on the putative function of the proteins found in the UniProt and GenBank databases. The classification of the identified proteins according to their involvement in biological processes resulted in twenty categories, with nine highly represented categories (Supplementary Figure S4D). One of the main categories was the developmental process, with more than 1000 of the identified proteins belonging to this group.

**Figure 6:**
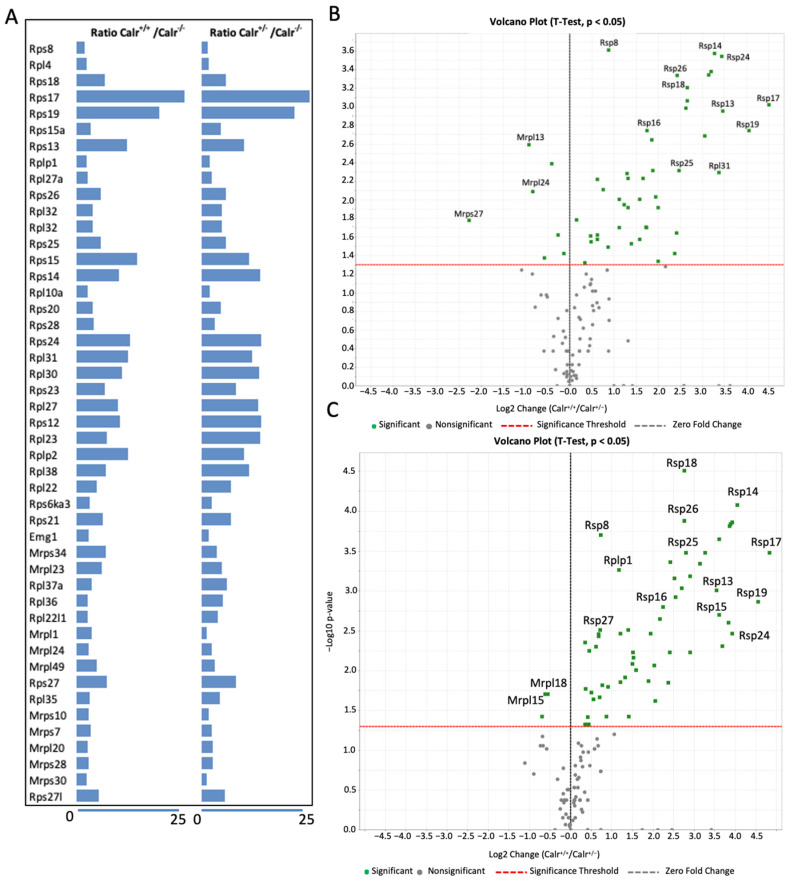

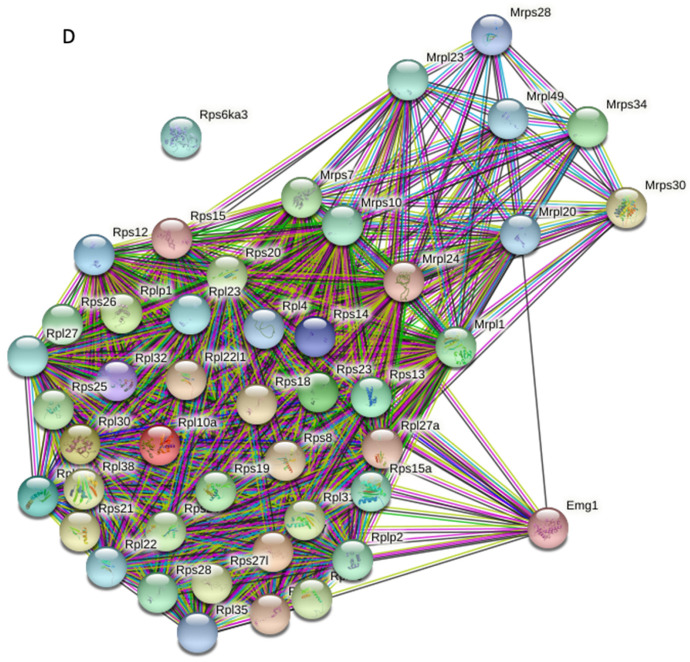
(**A**): The mass spectrometric based label free quantification permitted the identification of the ribosomal proteins found to be down-regulated in Carl^−/−^ embryonic kidney. (**B**,**C**): Volcano plots illustrating the ribosomal proteins found to be up-regulated in Calr^+/+^ compared to Calr^−/−^ (**B**) and in Calr^+/−^ compared to Carl^−/−^ (**C**). Remarkably, several mitochondrial ribosomal proteins were up-regulated in Calr^−/−^. (**D**): Networking analysis of ribosomal protein found to be regulated in the Calr^−/−^ compared to Calr^+/+^ and Calr^+/−^ using the protein networks software String (https://string-db.org, accessed on 24 March 2021). A strong interaction node was established between the proteins supporting common functional processes.

Since protein–protein interactions are the key mechanisms for almost all biological processes including development, the extraction of information on possible protein–protein interactions and the pathway regulation connecting the regulated proteins in Calr^+/−^ and Calr^−/−^ embryonic kidney might deliver significant information on the processes that are impaired under Calr deficiency conditions. For this purpose, we investigated the interaction networks between the regulated proteins using STRING 11.0 (http://string.embl.de, accessed on 24 March 2021). Further analysis of proteins expressed solely in Calr^+/+^ and Calr^+/−^ revealed two strong interaction nodes from proteins involved in two main processes, which are RNA-metabolism and oxidative phosphorylation (Supplementary Figure S5A). This suggests that the Calr^−/−^ embryonic kidney may suffer from an abnormality in RNA-metabolism and energy shortage. The investigation of the interaction networks between the proteins overexpressed in Calr^+/+^ and Calr^+/−^ compared to Calr^−/−^ strongly supports our assumptions because the networks show three strong interaction nodes. Two of the nodes accumulated proteins involved in RNA-metabolism and oxidative phosphorylation, while the third node involves ribosomal proteins and revealed disturbance in ribosome formation and protein turnover (Supplementary Figure S5B). A close examination of the ribosomal proteins found to be regulated showed significant alteration of proteins from both large and small ribosomal subunits and this revealed the severe disturbance in ribosomal biogenesis and protein synthesis (Figure 6A–D).

### 2.5. Calr Knockout Results in an Alteration in Ribosomal Protein Expression

**Figure 7:**
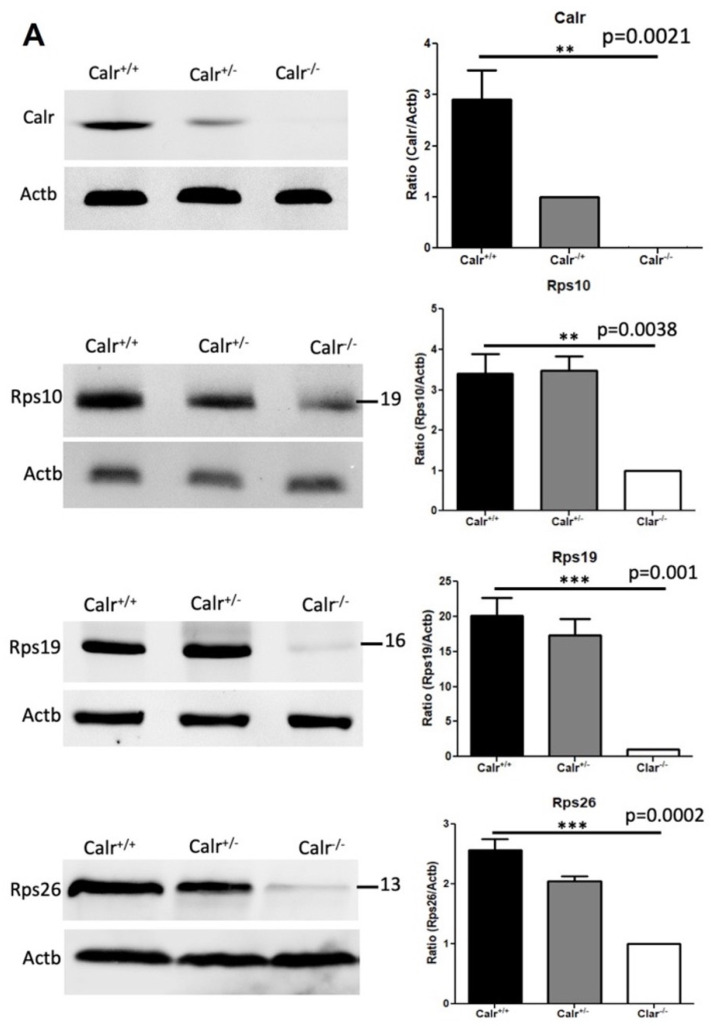

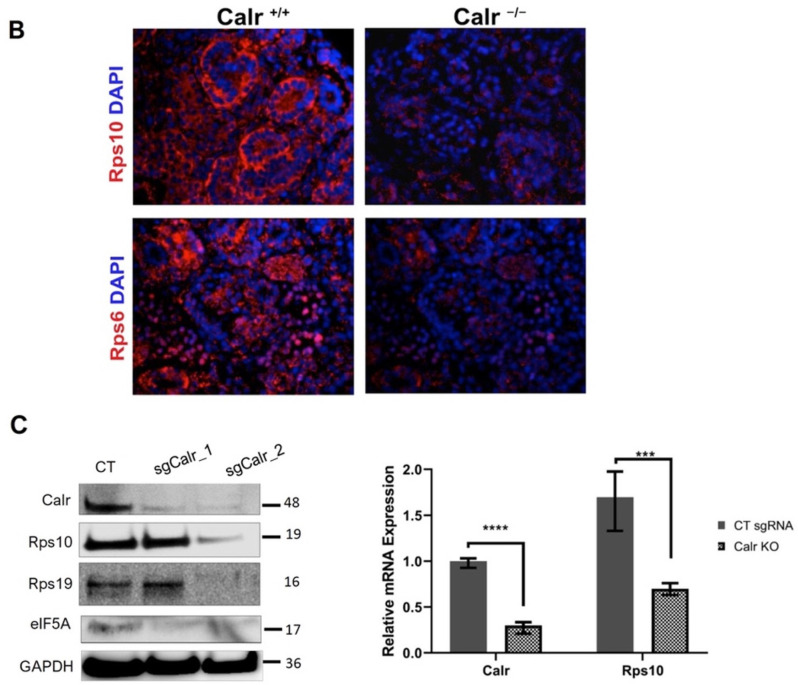
Calr deficiency is associated with down-regulation in ribosomal protein expression. (**A**): Western blot analyses of ribosomal proteins Rps10, Rps19, and Rps26 in protein extracts from Calr^+**/+**^**,** Calr^+/−^, and Calr^−/−^ embryonic kidneys. Embryonic kidneys were harvested from three different pregnant Calr^+/-^ mice. After genotyping, embryos from the same mother and genotype were grouped together and their embryonic kidney protein extracts were pooled together. After protein estimation Western blot analyses were performed in triplicate for each investigated protein. For the comparative analysis of the samples, the one-way ANOVA was used. The results are presented as the mean ± s.d. from at least three independent experiments. Differences were considered statistically significant when *p* < 0.05. (**B**): Immunofluorescence staining against Rps10 and Rps6 in Calr^+/+^ and Calr^−/−^ embryonic kidney tissue sections with a magnification of 20×. (**C**): Western blot analysis of protein extract from MDCK cells (left) and mRNA quantification after the knockout of Calr in MDCK cells (right). Calr was knocked out using the CRISPR/cas9 system with two different sgRNAs. Western blots were probed with Calr, GAPDH, Rps10, Rps19, and eIF5a antibodies. Calr and Rps10 mRNA quantifications in the sgCalr-2 sample were carried out using qPCR. Down-regulation of Calr using the CRISPR/cas9 system revealed an association between Calr deficiency and alteration in ribosomal protein expression. Significant differences: (**) *p* < 0.01, (***) *p* < 0.001, (****) *p* < 0.0001.

The transcriptomic and proteomic data analyses showed a significant alteration in the expression of ribosomal proteins in Calr^−/−^ mice embryonic kidneys. Western blot analysis and MS quantification of embryonic kidney protein extracts from the three genotypes confirmed the significant down-regulation of Rps10, Rps19, and Rps26 kidneys (Figure 7A) and Rps12, Rps13, Rps14 Rps15, Rps17, and Rps19 (Supplementary Figure S6A,B) in Calr^−/−^. Moreover, the staining of embryonic kidney sections confirmed the extent of the alteration in the expression of the ribosomal proteins; no Rps10 or Rps6 could be detected in Calr^−/−^ embryonic kidney sections (Figure 7B). In order to investigate the link between Calr knockout and ribosomal protein expression, we established an in vitro Calr knockout model in MDCK renal tubule cells using the CRISPR/cas9 endonuclease system. The western blot analyses confirmed dose dependent down-regulation of Calr expression in MDCK cells and demonstrated a significant depletion of the 40S ribosomal subunit encoding proteins Rps10 and Rps19, which revealed a disturbance in ribosome biogenesis. Moreover, the expression of essential components for the protein synthesis, e.g., elongation, revealed that the initiation factor eEIF5a significantly decreased in the Calr knockout MDCK cells (Figure 7C).

The Calr knockout was accompanied by significant morphological, proteomic and transcriptomic alterations. Our in vivo and in vitro investigations further highlighted that these aberrations were accompanied by a significant drop in ribosome biogenesis. The decrease in ribosomal protein expression in the Calr knockout kidney was not limited to embryonic stages but could also be demonstrated in cells derived from adult kidneys, revealing a potential role of Calr in ribosomal biogenesis.

## 3. Discussion

The kidneys develop through branching morphogenesis which is a process that involves complex growth and differentiation processes and is driven by the signals coming from the surrounding cells in the nascent mesenchymal compartment [16]. During the past years, several key genes and pathways in kidney development have been identified. The trophic factors, especially GDNF, bone morphogenetic proteins (BMPs), and fibroblast growth factors (FGFs) are involved in renal growth and differentiation. They govern the signaling involved in the UB branching morphogenesis as well as in the maintenance and differentiation of the nephrogenic mesenchyme in the embryonic kidney [17]. Calr knockout causes embryonic lethality due to dysfunctional cardiac development [15]. Increases in the cytoplasmic Ca^2+^ concentration is required in normal cardiomyocytes to drive cardiac myofibrillogenesis. This indispensable change in the Ca^2+^ concentration depends on Calr and is absent under Calr deficiency conditions [18]. The functional role of the ER chaperone and calcium binding protein Calr in nephrogenesis has not yet been explored. In order to investigate the potential role of Calr in embryonic kidney development and the impact of Calr deficiency on nephrogenesis, we performed a comparative transcriptomic and proteomic analysis. Overall, Calr deficiency resulted in the retardation of kidney development and in significant transcriptome and proteome alterations. Moreover, our data revealed that Calr deficiency triggered severe disorders in nephrogenesis pathways as significant expression alterations of Wnt signaling key proteins (e.g., Wnt7a, Wnt11, Wnt10b, Fzd10, Fzd9, Prkcb, Prkcq, and Kremen2) were identified. The inductive signaling between ureteric bud epithelium and metanephric mesenchyme is mainly regulated by Wnt feedback signaling [19,20]. While Calr is a major part of cellular calcium homeostasis, it is simultaneously an essential ER chaperone for the folding and trafficking of glycoproteins and proteins involved in cell signaling and gene expression [21]. The knockout of Calr could result in the disruption of protein folding and ER stress, which causes disturbance in translational machinery. This may explain the observed retardation in kidney development in homozygous mice.

One of the surprising and interesting aspects highlighted by our data is the alteration in the expression of ribosomal proteins. We demonstrated an almost complete depletion of several proteins of the 40S and 60S ribosomal subunits. Moreover, our in vitro experiments confirmed the correlation between the downregulation of Calr and the impairment of ribosomal protein expression. Ribosome biogenesis is well organized and undergoes strict regulation. Emerging studies have demonstrated that the ribosome plays an important role not only in normal cell physiology but also in reaction to stimuli and in pathogenesis of diseases. Moreover, ribosome biogenesis controls the cell growth and the proliferation and alterations in ribosomes are reflected in the aberration of cell proliferation, cell cycle arrest, apoptosis, and pathological manifestation [22,23].

In addition to their role in ribosome biogenesis, ribosomal proteins have been found to exercise diverse extra ribosomal functions, e.g., in cell growth and proliferation, in DNA-repair, and in cellular differentiation and development [24,25,26,27,28,29,30,31]. Impairment of ribosomal protein functions were associated with hematological and metabolic disorder and might result in cardiovascular diseases and cancer [32,33,34,35,36]. Mutation in genes coding for ribosomal proteins are associated with erythropoiesis abnormality resulting in clinical syndromes, e.g., Diamond–Blackfan anemia (DBA) [37] and 5q-syndrome [38]. Mutations in the RPS10 are associated with Diamond–Blackfan anemia and 30% of these patients have horseshoe or sigmoid kidneys [39]. Interestingly, the Diamond–Blackfan patients have a higher chance of developing myelodysplastic syndrome, which is associated with the Calr gene exon 9 mutation [40,41,42]. This remodeling of the translational machinery causes massive aberrations in the developmental process and affects not only the urogenital but also the circulatory and reproductive systems, eventually causing the embryonic lethality upon calreticulin deficiency. Our data revealed a disturbed ribosome biogenesis in the Calr^−/−^ embryonic kidney and highlighted a substantive role of Calr in protein biogenesis. We believe that a better understanding of the relationships between Calr deficiency and ribosomal protein expression alteration would provide new insights for understanding how Calr impacts the ribosome biogenesis and the embryonic kidney development and allow novel views on the processes governing organ development.

## 4. Materials and Methods

### 4.1. Animals

Calreticulin heterozygous (Calr^+/−^) and wildtype (Calr^+/+^) littermate mice in identical C57BL/6J genetic backgrounds were obtained from Professor Marek Michalak, University of Alberta, Edmonton, Alberta, Canada. Mice were bred under specific pathogen-free housing conditions and genotyped as previously described in Michalak et al. [15]. For our study, pregnant Calr^+/−^ mice were sacrificed at different stages of embryonic development (embryonic days 13.5: E13.5; 14,5: E14.5; and 16.5: E16.5), the embryos were harvested, and the kidneys were dissected. For genotyping, a piece of the embryo tail was used. The kidneys were further prepared for either histochemical staining or transcriptomics or proteomics analysis. All experimental procedures were performed in accordance with the German animal care and ethics legislation (NIH standards) and were approved by the local Ethics Committee of the University Medical Center Göttingen, Germany (33.14-42502-04-11/0598).

### 4.2. Histochemical Staining

For the histological staining of the embryonic kidneys, the excised kidneys were fixed overnight in 4% buffered formaldehyde solution and subsequently embedded in paraffin blocks. The paraffin embedded sections were deparaffinized and rehydrated and stained with hematoxylin solution Gill III and eosin Y solution (Merck, Darmstadt, Germany) according to the manufacturer’s protocol.

### 4.3. Ex-Vivo Organ Culture of Embryonic Kidney

The ex vivo organ culture was carried out according to Davies et.al [43]. Pregnant mice were sacrificed at stage E13.5 of embryonic development, embryos were harvested, and the kidneys were dissected. After genotyping, 6 kidney rudiments from every genotype (Calr^+/+^, Calr^+/−^, and Calr^−/−^) were plated on a transparent PET membrane with 0.4 µM pore size (24 well, BD Falcon). The membrane was placed in one well of a 24 well plate, containing 400 µL kidney culture medium (KCM, DMEM, 10% inactivated FCS). This enabled the kidney to contact the medium without drowning it. Under these conditions, it was possible to keep the kidney cultured at 37 °C and 5% CO_2_ for at least 144 h.

### 4.4. Immunofluorescence Staining of the Cultured Kidneys and Immunohistological Analysis of the Kidney Sections

The embryonic kidneys were cultured ex vivo for 72 h, thereafter, the kidney rudiments were fixed in cold methanol at −20 °C for 30 min. The fixation step was followed by 3 washing steps each of which were for 5 min in PBS. The primary antibody rabbit monoclonal anti-laminin antibody (Sigma-Aldrich, St Louis, MO, USA) was diluted in PBS and incubated at 4 °C overnight. The cultured kidney rudiments were washed in PBS for 4 h and the secondary antibody (Molecular Probes Alexa Fluor 555 goat anti-rabbit IgG (1:500)) was added overnight at 4 °C. The UB was stained using lectin Dolichos biflorus agglutinin (DBA) for at least 3 h. After incubation, the kidneys were washed in PBS for 3 h and embedded for microscopic analysis.

Immunostaining of deparaffinized and rehydrated embryonic kidney sections was performed to detect the expression and distribution of several proteins. The sections were treated with an antigen retrieval solution (1.8 µM citric acid and 8.2 µM sodium citrate) prewarmed in a food steamer for 25 min. The sections were blocked with 10% goat serum in PBS for 1 h an incubated overnight at 4 °C with the primary antibodies (The following primary antibodies were used: rabbit monoclonal anti-Calbindin-1, anti-Wt1, anti-Six2, anti-Pax2, anti-Calr (Abcam, Cambridge, UK), rabbit monoclonal anti-Rps6, anti-Rps10 (Invitrogen), and rabbit monoclonal anti-Sod1 antibody (Abnova, Taipei, Taiwan). The primary antibodies were detected with fluorescence labeled secondary antibodies (Molecular Probes Alexa Fluor 555 goat anti-mouse IgG antibody and Alexa Fluor 555 goat anti-rabbit IgG) for 1 h at room temperature. For negative controls, tissue sections were incubated only with the secondary antibody. The slides were mounted with coverslips in Vectashield mounting medium with DAPI to counterstain the nuclei (Vector Laboratories, Burlingame, CA, USA).

### 4.5. Cell Culture

Madin–Darby canine kidney (MDCK) cells were obtained from American Type Culture Collection (ATCC) and propagated in Dulbecco’s Modified Eagle’s Medium (DMEM), 10% fetal bovine serum (FBS), 1% Penicillin/Streptomycin, and 1% L-Glutamine at 37 °C in a 5% CO_2_-atmosphere. Cells are cultured in T25 flasks and were split twice in a week. For cell passaging, MDCK cell were trypsinized for a short period of time to avoid alteration of cell structure.

### 4.6. Generation of Knockout Cells

The sgRNA sequences of Calr (XM8622117) for the canis lupus species were designed using the online tool BlueHeronBio (Origene, Herford, Germany). The sgRNA sequence 5′ GTAGATGGCGGGTTCGGCAG 3′ was inserted into the pLenti-Cas-Guide plasmid (Addgene plasmids #3931646) with *BamHI* and *BsmBI* restriction enzymes to generate the pLenti-Cas9-Calr construct was later confirmed by Sanger sequencing.

In order to knockout Carl in MDCK cells, pLenti-Cas9-Calr transient transfection was performed. One day before the transfection, 200,000 cells/mL was seeded in 6 well plates with MEM medium. Prior to the transfection, the culture medium was changed to FCS-free Opti-MEM medium. Lipofectamine 2000 (Invitrogen, Carlsbad, CA, USA) was used to transfect the cells. Lipofectamine plasmid DNA mix was prepared with Opti-MEM based on the instructor’s protocol. For 6 well plate transfection, 4 μg plasmid DNA was added to 250 μL Opti-MEM medium separately and incubated for 10 min at room temperature. Subsequently, the DNA mixture was added to the Lipofectamine 2000 mixture and incubated for 30 min before being added to the cells. The transfection success was assessed using Western blot.

### 4.7. Transcriptome Analysis

For transcriptome investigations, 3 pregnant Calr^+/−^ mice were used. Embryos (8–10/pregnant mice) were harvested and the kidneys were isolated. After genotyping, kidneys from the same genotype and same mother were pooled together and processed for RNA extraction. Total RNAs were isolated from Calr^+/+^, Calr^+/−^, and Calr^−/−^ embryonic kidneys using the Trizol (Invitrogen) method according to the manufacturer’s recommendations. The samples were then treated with DNAse I (Sigma) to remove the DNA contamination. The quality of RNA was ascertained using the Agilent 2100 Bioanalyzer (Agilent Technologies, Santa Clara, CA, USA) microfluidic electrophoresis. For sequencing, the RNA samples were prepared using the “TruSeq RNA Sample Prep Kit” according to the manufacturer’s protocol (Illumina, San Diego, CA, USA). Single read (50 bp) sequencing was conducted using a HiSeq 2000 (Illumina, San Diego, CA, USA). The sequences were aligned to the genome reference sequence of *Mus Muscullus* (Ensembl genome assembly 3.2.1; https://www.ensembl.org/info/genome/genebuild/assembly.html, accessed on 24 March 2021) using the STAR alignment software (Cold Spring Labarotory, Cold Spring Harbor, USA) (version 2.3.0e, https://github.com/alexdobin/STAR, accessed on 24 March 2021) [44] allowing for 2 mismatches within 50 bases. SAMtools package(version 0.1.18) and HTSeq (version 0.6.1p1) were used for the filtering of unique hits and counting [45,46]. Candidate genes were filtered to a minimum of 2-fold change and FDR-corrected *p*-value < 0.05. Gene annotation was performed using *Mus Muscullus* from Ensembl v78 (www.ensembl.org, accessed on 1 March 2019) via the biomaRt package (version 2.24.0) [47]. GO enrichment analysis for candidate genes was conducted with the Goseq package (version 1.2) [48] using standard parameters.

### 4.8. Kidney Lysis, Protein Extraction and Two -Dimensional Gel Electrophoresis (2-DE)

For 2D gel electrophoresis, embryos from 6 pregnant Calr^+/−^ females (8–10 embryo/female) were used. For the protein extraction for 2D gel electrophoresis (2-DE), the kidneys from embryos at the same embryonic stage and from the same genotype and female (8–10 embryos/pregnant female) were pooled, disrupted with a lysis buffer (9.5 M urea, 2% CHAPS (*w/v*), 2% ampholytes (*w/v*), 1% DTT), and vortexed. After adding the lysis buffer, the samples were incubated at 4 °C for 30 min. To remove the cell debris, centrifugation was carried out at 13,000× *g* and 4 °C for 30 min. The supernatant was recentrifuged for an additional 30 min at 13,000× *g* and 4 °C to get maximal purity. The supernatants were collected and the pellets were discarded and the resulting samples were used immediately or stored at –80 °C until use.

In order to reduce the salt contamination and to enrich the proteins, methanol–chloroform precipitation according to Wessel and Flügge [49] was performed. The pellet was dried and dissolved in the lysis buffer. The total protein concentration was determined using the Bio-Rad protein assay (Bio-Rad, Hercules, CA, USA) according to Bradford. BSA (Sigma, Steinheim, Germany) was used as a standard. In order to guarantee experimental reproducibility, we generated triplicate gels from each kidney pool.

The 2D protein separation was carried out as previously described [50]. The 2-DE gels were stained with Flamingo fluorescent gel stain (Bio-Rad, Hercules, CA, USA) following the manufacturer instructions. After staining, the gels were scanned at 50 µm resolution on a Fuji FLA-5100 scanner. The digitalized images were analyzed using Delta 2D 3.4 (Decodon, Braunschweig, Germany). For protein visualization, the 2-DE gels were additionally stained overnight with colloidal Coomassie blue, Roti-Blue (Roth, Karlsruhe, Germany).

### 4.9. Mass Spectrometric Analysis and Protein Identification

Significantly regulated spots were excised from the gels and tryptic in-gel digestion and peptide extraction were performed as previously described by Dihazi et al. [51]. Briefly, gel spots were rinsed twice in 25 mM ammonium bicarbonate (amBic) and once in water, shrunk with 100% acetonitrile (ACN) for 15 min, and dried in a SpeedVac (Thermo Fisher Scientific, Waltham, MA, USA) for 20–30 min. All excised spots were incubated with 12.5 ng/μL sequencing grade trypsin (Roche Molecular Biochemicals, Basel, CH) in 25 mM amBic overnight at 37 °C. Peptide extraction was carried out twice by firstly using 50% ACN/1% trifluoroacetic acid (TFA) and then 100% ACN. All extracts were pooled and the volume was reduced using SpeedVac. Tryptic peptides were subjected to mass spectrometric sequencing using a Q-TOF Ultima Global mass spectrometer (Micromass, Manchester, UK) equipped with a nanoflow ESI Z-spray. Protein identification was carried out with the Mascot search engine against MSDB and Swissprot databases by using a peptide mass tolerance and fragment tolerance of 0.5 Da.

### 4.10. Protein Extraction, SDS-PAGE, in Gel Tryptic Digestion, and Mass Spectrometric Analyses

For mass spectrometric quantification of the protein alteration in the Calr^−/−^ kidney, the samples from the same genotype and same mother were pooled together. In order to generate enough pools and to assure biological and experimental replication, we used 6 different pregnant Cal^+/−^ with 8–10 embryos/mice. The embryonic kidneys from the three genotypes were harvested from the embryos and the protein extraction was carried out using the lysis buffer as described above. Protein extracts were separated by SDS-PAGE, the gels were stained with Coomassie Blue, each lane excised and cut into 20 slices of equal size, and slices subjected to in-gel digestion with trypsin. For mass spectrometric analysis, samples were enriched on a self-packed reversed phase-C18 precolumn (0.15 mm ID × 20 mm, Reprosil-Pur120 C18-AQ 5 µm, Dr. Maisch, Ammerbuch-Entringen, Germany) and separated on an analytical reversed phase-C18 column (0.075 mm ID × 200 mm, Reprosil-Pur 120 C18-AQ, 3 µm, Dr. Maisch) using a 30 min linear gradient of 5–35% acetonitrile/0.1% formic acid (*v:v*) at 300 nl min-1). The eluent was analyzed on a Q Exactive hybrid quadrupole/orbitrap mass spectrometer (ThermoFisher Scientific, Dreieich, Germany) that was equipped with a FlexIon nanoSpray source and operated under the Excalibur 2.4 software using a data-dependent acquisition method. Each experimental cycle was of the following form: one full MS scan across the 350–1600 m/z range was acquired at a resolution setting of 70,000 FWHM and AGC target of 10^6^ and a maximum fill time of 60 ms. Up to the 12 most abundant peptide precursors of charge states from 2 to 5 above a 2 × 10^4^ intensity threshold were then sequentially isolated at a 2.0 FWHM isolation width, fragmented with nitrogen at a normalized collision energy setting of 25% and the resulting product ion spectra was recorded at a resolution setting of 17,500 FWHM and there was an AGC target of 2 × 10^5^ and a maximum fill time of 60 ms. The selected precursor m/z values were then excluded for the following 15 s. Two technical replicates per sample were acquired.

Peaklists were extracted from the raw data using Raw2MSMS software v1.17 (Max Planck Institute for Biochemistry, Martinsried, Germany). Protein identification was achieved using the MASCOT 2.5.1 software (Matrixscience, London, UK). Proteins were identified against the UniProtKB mouse reference proteome v2017.09 (16930 protein entries) along with a set of 51 contaminants commonly identified in our laboratory. The search was performed with trypsin as the enzyme and iodoacetamide as the cysteine blocking agent. Up to two missed tryptic cleavages and methionine oxidation as a variable modification were permitted. Search tolerances were set to 10 ppm for the precursor mass and 0.05 Da for the fragment masses and ESI-QUAD-TOF was specified as the instrument type.

Scaffold software version 4.8.9 (Proteome Software Inc., Portland, OR, USA) was used to validate MS/MS based peptide and protein identifications. Peptide identifications were accepted if they could be established at greater than 95.0% probability by the Percolator algorithm. Protein identifications were truncated to a false discovery rate of 1% at a minimum of 2 confidently identified peptides. Protein hits that contained similar peptides and, further, could not be differentiated based on MS/MS analysis alone were grouped to satisfy the principles of parsimony. Proteins sharing significant peptide evidence were grouped into clusters. Protein abundances were estimated by their Spectral Counts following total sums normalization between all replicates.

### 4.11. Western Blot Analysis

Western blot analyses were performed according to Towbin et al. [52]. Equal amounts of proteins (50–75 μg) were separated by polyacrylamide gel electrophoresis (SDS-PAGE) and transferred on nitrocellulose membranes (Amersham Pharmacia Biotech, Buckinghamshire, UK). The membranes were blocked in 5% non-fat dry milk in a TBST-buffer (20 mM Tris-HCl, pH 7.4 150 mM NaCl 0.1% Tween 20) and incubated with primary antibodies (anti-Calr, anti-Rps10, anti-Rps19, anti-Rps26, and anti-eIF5A) overnight at 4 °C. In order to visualize the protein bands, fluorescence labeled secondary antibodies were used. In order to confirm equal protein loading, the blots were treated with anti-Actb or anti-GAPDH antibodies (Sigma, Taufkirchen, Germany).

### 4.12. Bioinformatics

The classification of the identified proteins according to their mainly known and postulated functions was carried out using DAVID bioinformatics (http://david.abcc.ncifcrf.gov, accessed on 21 February 2019) [53,54]. Gene symbols were used to investigate and categorize the gene ontology (GO) annotations (biological processes and molecular functions). Network analysis of known and predicted protein–protein interactions of the identified proteins was performed using STRING (search tool for the Retrieval of Interacting Genes/Proteins) [55].

### 4.13. Statistical Analysis

For 2-DE, the digitalized images were analyzed and spot matching across gels and normalization was performed using Delta2D 3.4 (Decodon, Braunschweig, Germany). Delta2D computes a “spot quality” value for every detected spot. This value shows how closely a spot represents the “ideal” 3D Gaussian bell shape. Based on average spot volume ratio, spots whose relative expression was changed at least 2-fold (increase or decrease) in triplicate between the compared samples were considered to be significant. In order to analyze the significance of protein regulation, Student’s t-test was performed and statistical significance was assumed for P values less than 0.05.

All blots were quantified using the ImageJ software. The data were compiled with the software package GraphPad Prism, version 8 (San Diego, CA, USA). The software was used for graphical presentation and analysis by either Student’s t-distribution or one-way ANOVA. The results are presented as the mean ± s.d. from at least three independent experiments. Differences were considered statistically significant when *p* < 0.05. Ureteric bud tips and nephrons were counted manually and the data were compiled with the software package GraphPad Prism, version 8.

## Supplementary Materials

The following are available online at https://www.mdpi.com/article/10.3390/ijms22115858/s1.
