# Supplementary material for: Calreticulin Deficiency Disturbs Ribosome Biogenesis and Results in Retardation in Embryonic Kidney Development
Source: Int J Mol Sci. 2021 May 30;22(11):5858. doi: 10.3390/ijms22115858 (PMC8198291; doi:10.3390/ijms22115858)
Supplement: Supplementary file 1 [file ijms-22-05858-s001.zip › ijms-1197453-supplementary/ijms-1197453-Supplementary Figures.pdf]

Supplementary Materials

Calreticulin Deficiency Disturbs Ribosome Biogenesis and Results in Retardation in Embryonic Kidney Development

Nazli Serin [1,2,†], Gry Helene Dihazi [3,†], Asima Tayyeb [4], Christof Lenz [3,5], Gerhard A. Müller [1], Michael Zeisberg [1] and Hassan Dihazi [1,6,*]

[1] Clinic for Nephrology and Rheumatology, University Medical Centre Göttingen, Robert-Koch-Strasse 40, 37075 Göttingen, Germany; nazli.serin@med.uni-goettingen.de (N.S.); gmueller@med.uni-goettingen.de (G.A.M.); michael.zeisberg@med.uni-goettingen.de (M.Z.)
[2] University Medical Centre Göttingen, Department of Hematology and Oncology, University of Medical Center Göttingen, Robert-Koch-Strasse 40, 37075 Göttingen, Germany
[3] Institute for Clinical Chemistry/UMG-Laboratories, University Medical Centre Göttingen, Robert-Koch-Strasse 40, 37075 Göttingen, Germany; gryhelene.dihazi@med.uni-goettingen.de (G.H.D.); christof.lenz@med.uni-goettingen.de (C.L.)
[4] School of Biological Sciences, University of the Punjab Lahore, city post code, Pakistan; asima.sbs@pu.edu.pk
[5] Bioanalytical Mass Spectrometry, Max Planck Institute for Biophysical Chemistry, 37077 Göttingen, Germany
[6] Center for Biostructural Imaging of Neurodegeneration (BIN), University Medical Center Göttingen, 37075 Göttingen, Germany
* Correspondence: dihazi@med.uni-goettingen.de; Tel.: +049-551-39-91221; Fax: +049-551-39-91039
† These authors contributed equally to this work.

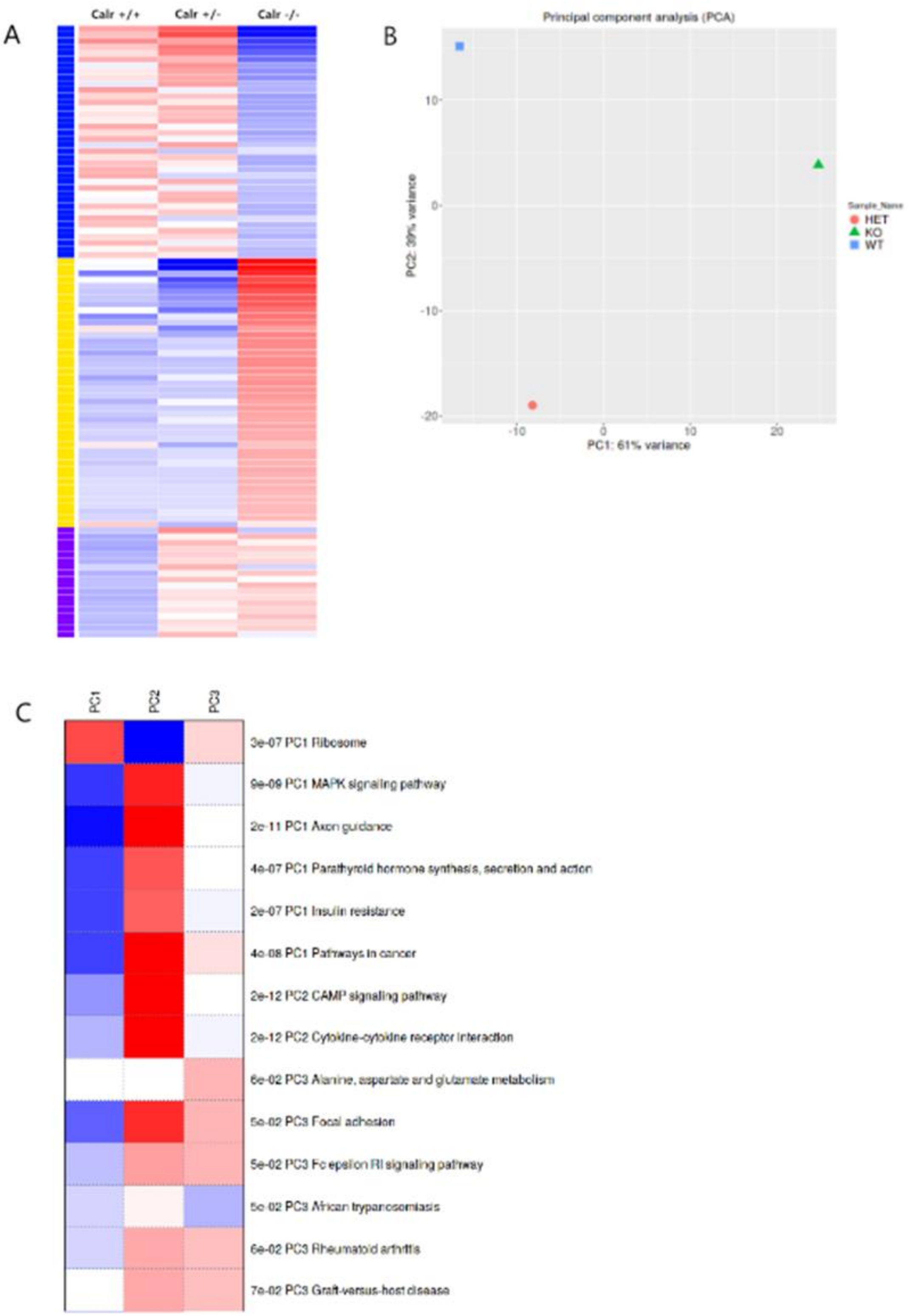

Supplementary Figure S1. Differential gene expression data analysis of the in vivo RNA sequence data. (**A**) K-means clustering is applied for clustering genes into groups based on their gene expression patterns across Calr$^{+/+}$,Calr$^{+/-}$, and Calr$^{-/-}$ samples. Firstly, genes are ranked by standard deviation and the top 1000 genes are used for clustering. (**B**) Principal component analysis (PCA) projecting three distinct genotypes on two dimensional space showing the variance between distinct groups represented in the x and y axes. (**C**) PCA loadings on gene expression data used to run pathway analysis with the PGSEA package. The adjusted p-values are used in order to rank the KEGG pathway enrichments for each of the three principal components.

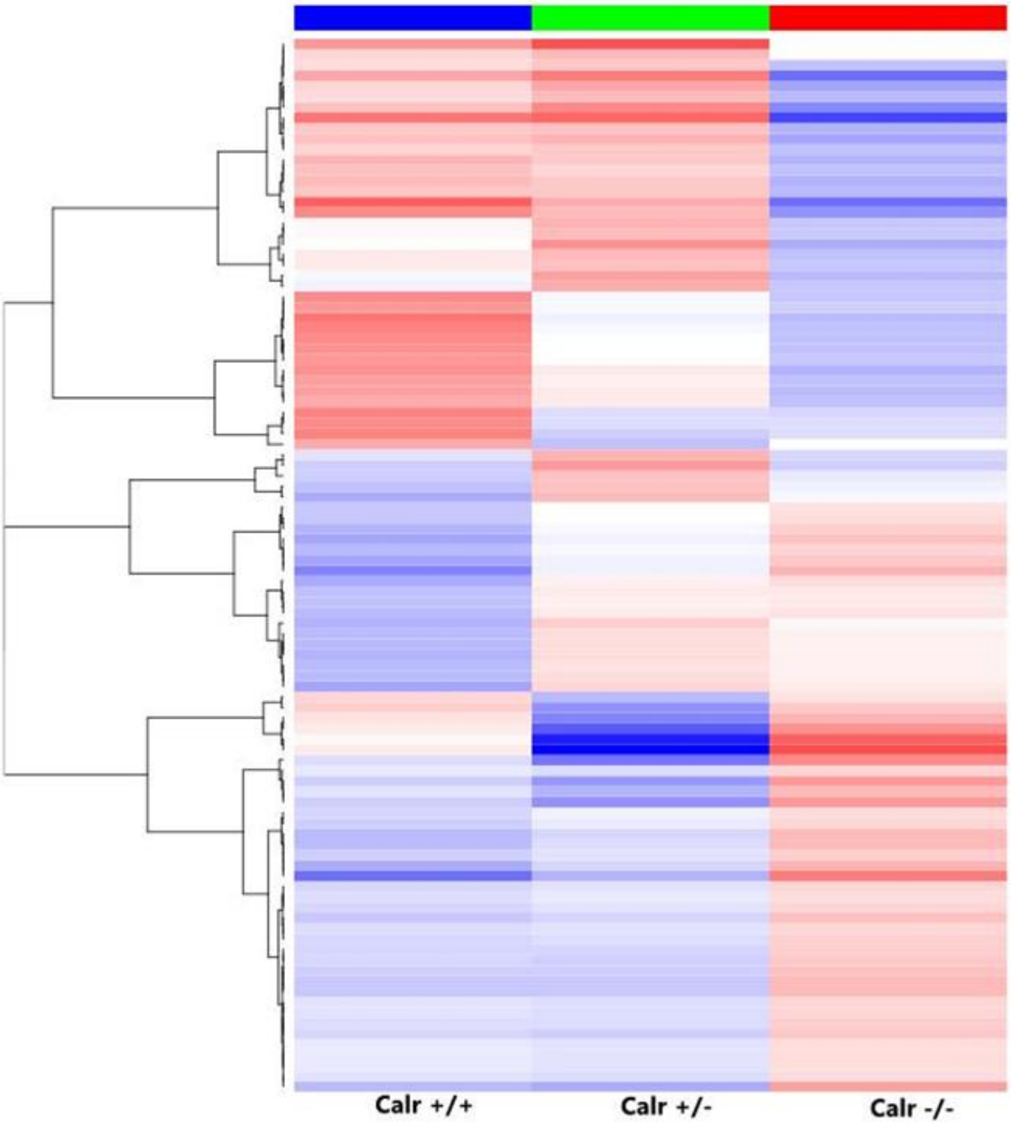

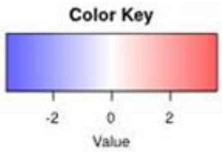

Supplementary Figure S2. Unbiased hiearchical clustering of in vivo gene expression profiles. Heat map of gene expression profiles shown with gene (row) to sample (column) clustering dendrograms. The colour corresponds to the square root of standardized TPM values scaled by the standard deviation and centred to the mean.

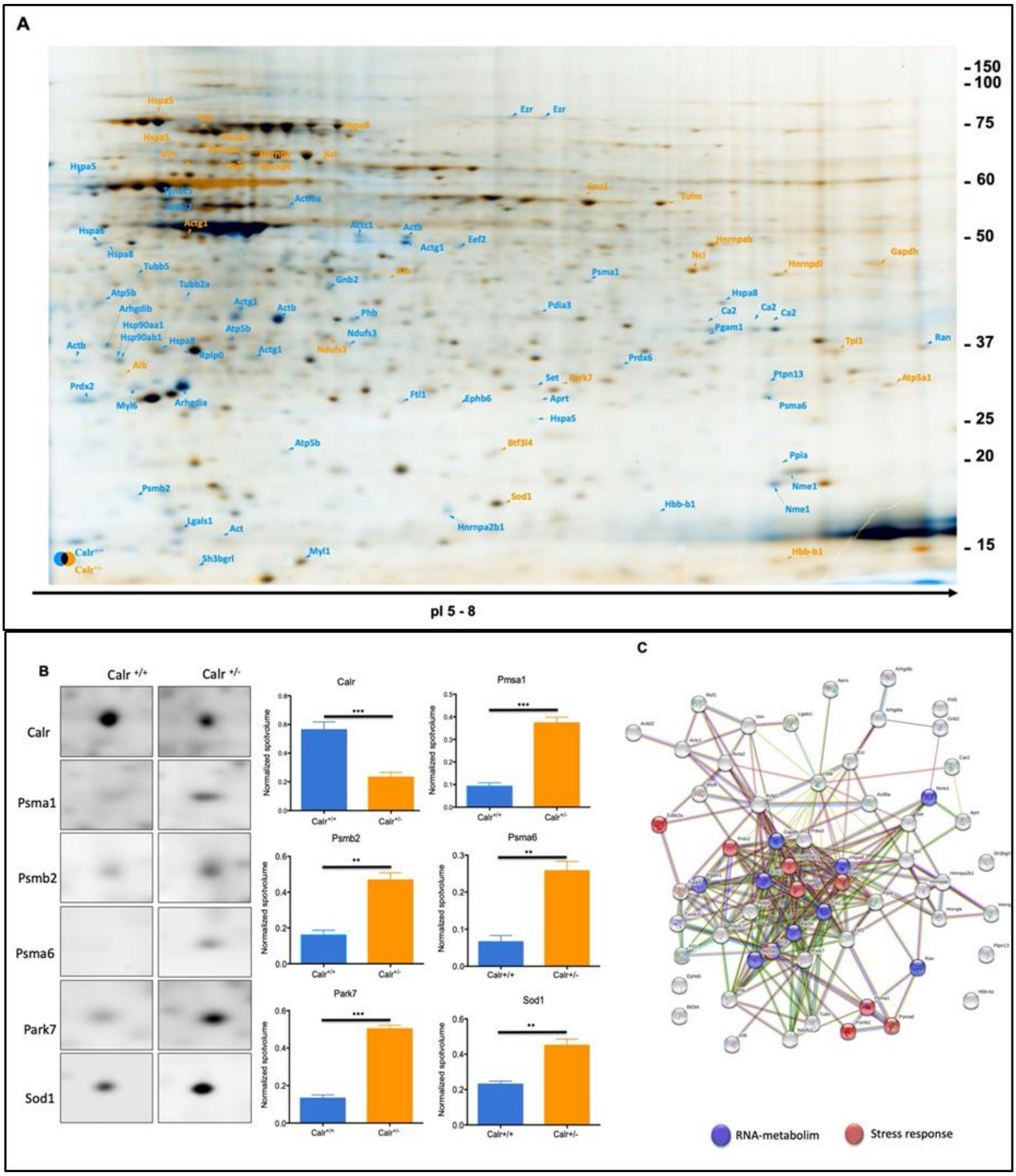

Supplementary Figure S3. 2-DE based comparative proteome analysis of the embryonic kidney from $Calr^{+/+}$ and $Calr^{+/-}$. (**A**) Overlapping 2-DE map of $Calr^{+/+}$ and $Calr^{+/-}$ embryonic kidney proteomes. Blue spots indicate higher expression in $Calr^{+/+}$ vs. $Calr^{+/-}$ embryonic kidney. Orange spots indicate an up-regulation of the proteins in $Calr^{+/-}$ embryonic kidney. Protein expressed in both phenotypes are overlapping spots and are shown in black. (**B**) Magnified images of the regions of interest showing differentially regulated proteins in the $Calr^{+/-}$ embryonic mouse kidney. The protein expression quantification for selected proteins is given in form of bar diagrams. Results are given as the means ± SD of the percentage volume of spot from at least three independent experiments ($p < 0.05$). (**C**) Interaction network of the protein found to be differentially expressed between the $Calr^{+/+}$ and $Calr^{+/-}$ embryonic kidneys.

A

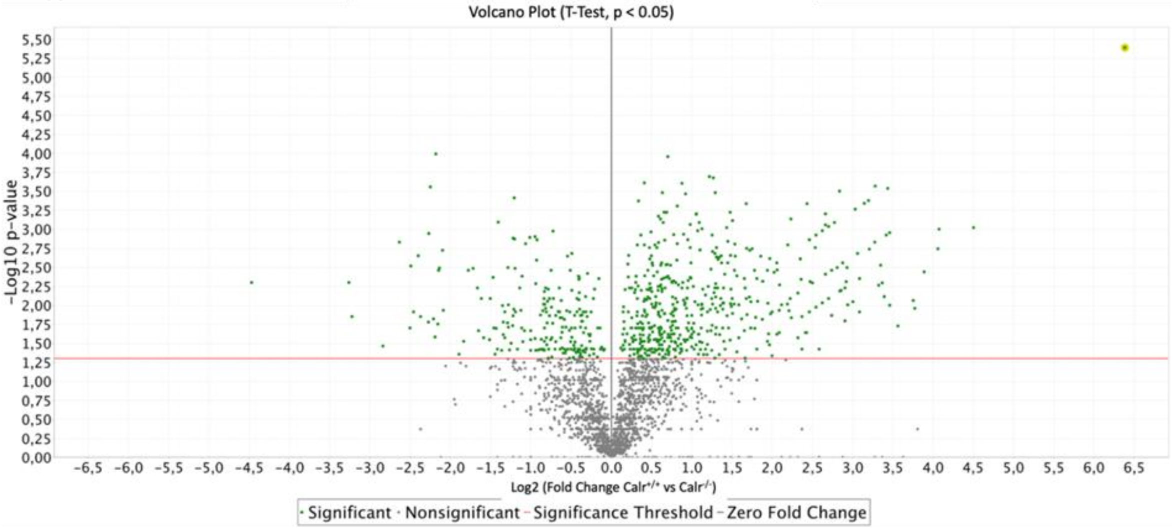
Volcano Plot (T-Test, p < 0.05)
−Log10 p-value
Log2 (Fold Change Calr+/+ vs Calr-/-)
Significant
Nonsignificant
Significance Threshold
Zero Fold Change

B

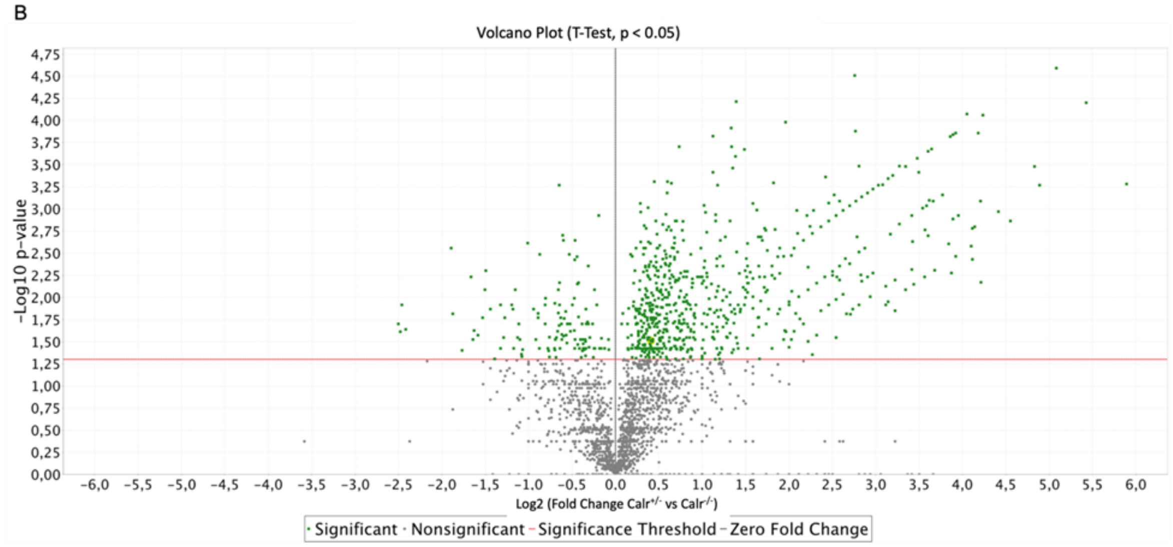
Volcano Plot (T-Test, p < 0.05)
−Log10 p-value
Log2 (Fold Change Calr+/- vs Calr-/-)
Significant
Nonsignificant
Significance Threshold
Zero Fold Change

C

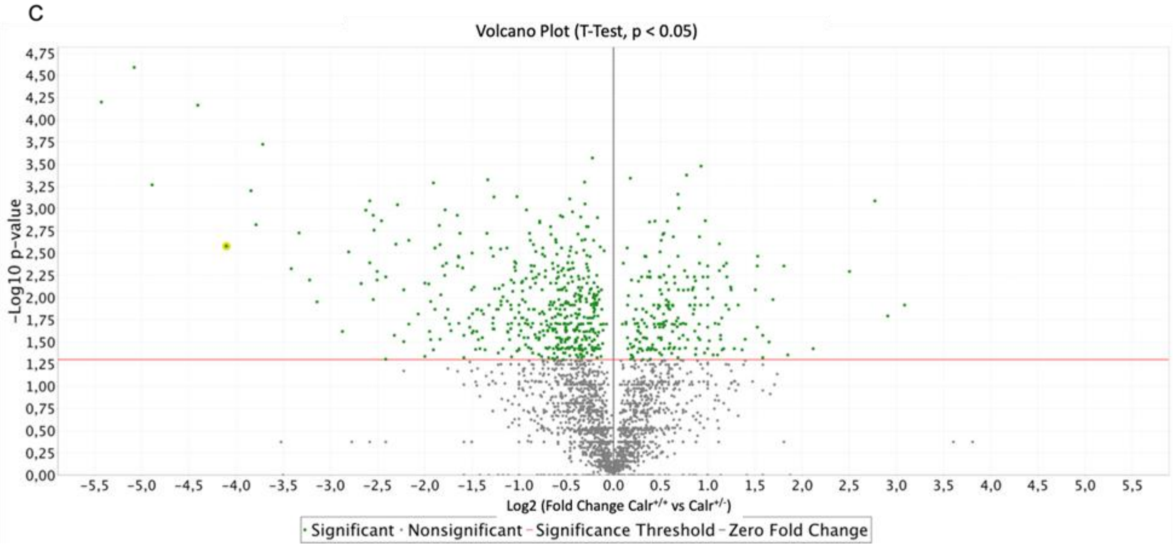
Volcano Plot (T-Test, p < 0.05)
−Log10 p-value
Log2 (Fold Change Calr+/+ vs Calr+/-)
Significant
Nonsignificant
Significance Threshold
Zero Fold Change

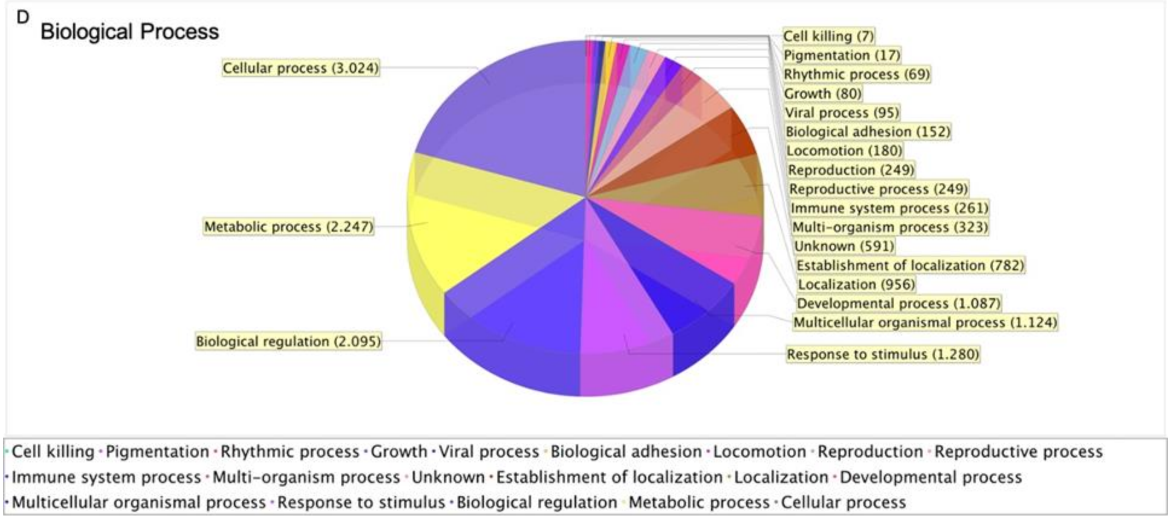

Supplementary Figure S4. (**A**–**C**) Volcano plot analysis of the identified and quantified proteins allowed the identification of proteins, which were differentially expressed between Calr$^{+/+}$ and Calr$^{-/-}$ (**A**), Calr$^{+/-}$ and Calr$^{-/-}$ (**B**), and Calr$^{+/+}$ and Calr$^{+/-}$ (**C**). (**D**) GO classification of the comparative proteome data. The categorization was achieved by correlating GO identification numbers corresponding to the biological process with the regulated proteins.

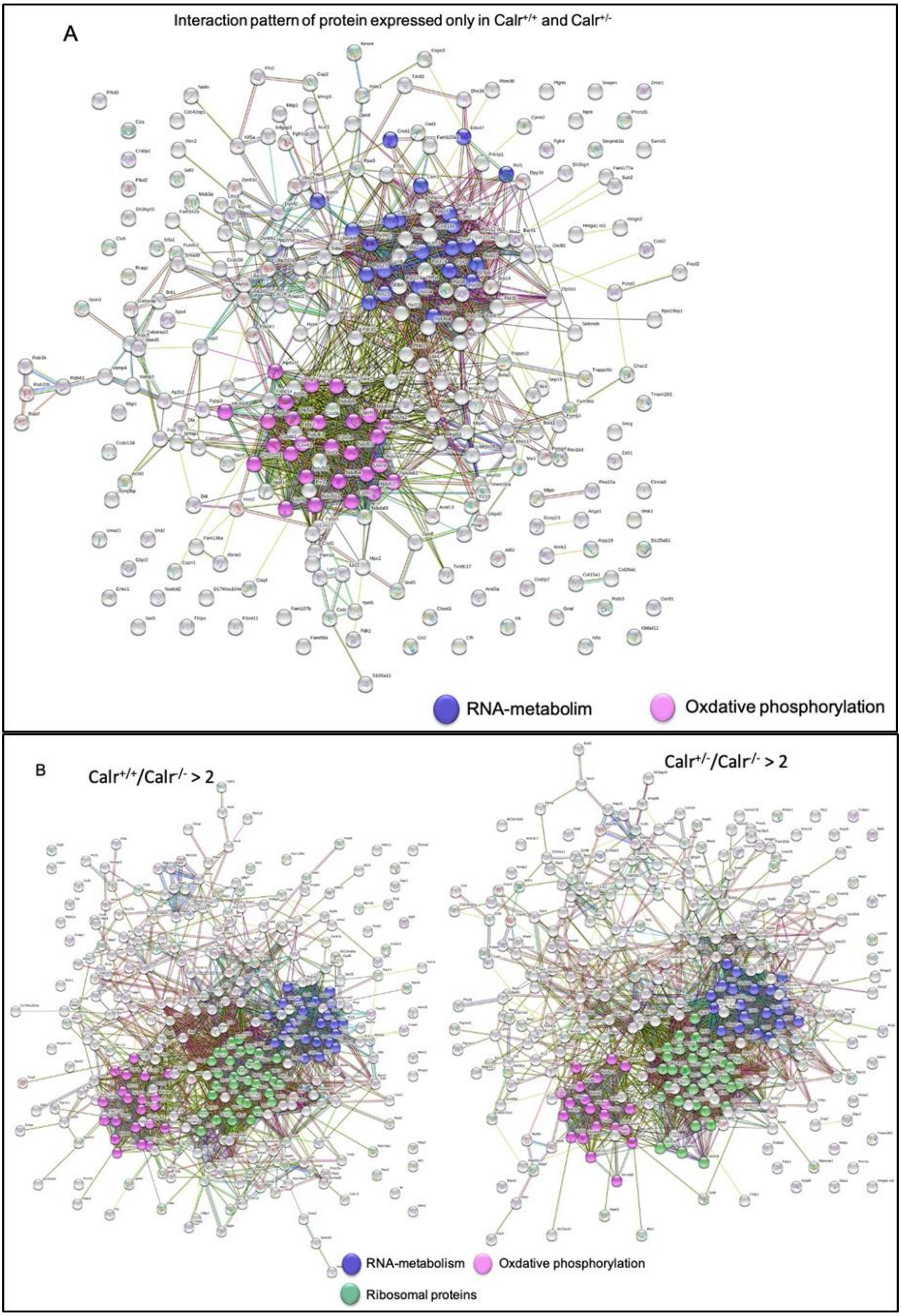

Supplementary Figure S5. String analysis of the comparative proteome data. (**A**) Protein interaction networks between the proteins found to only be expressed in Calr$^{+/+}$ and Calr$^{+/-}$ but not in Calr$^{-/-}$. (**B**) Protein interaction networks between the proteins found to be up-regulated in Calr$^{+/+}$ and Calr$^{+/-}$ compared to Calr$^{-/-}$ mouse kidneys. The networks were generated using the protein networks software string (https://string-db.org).

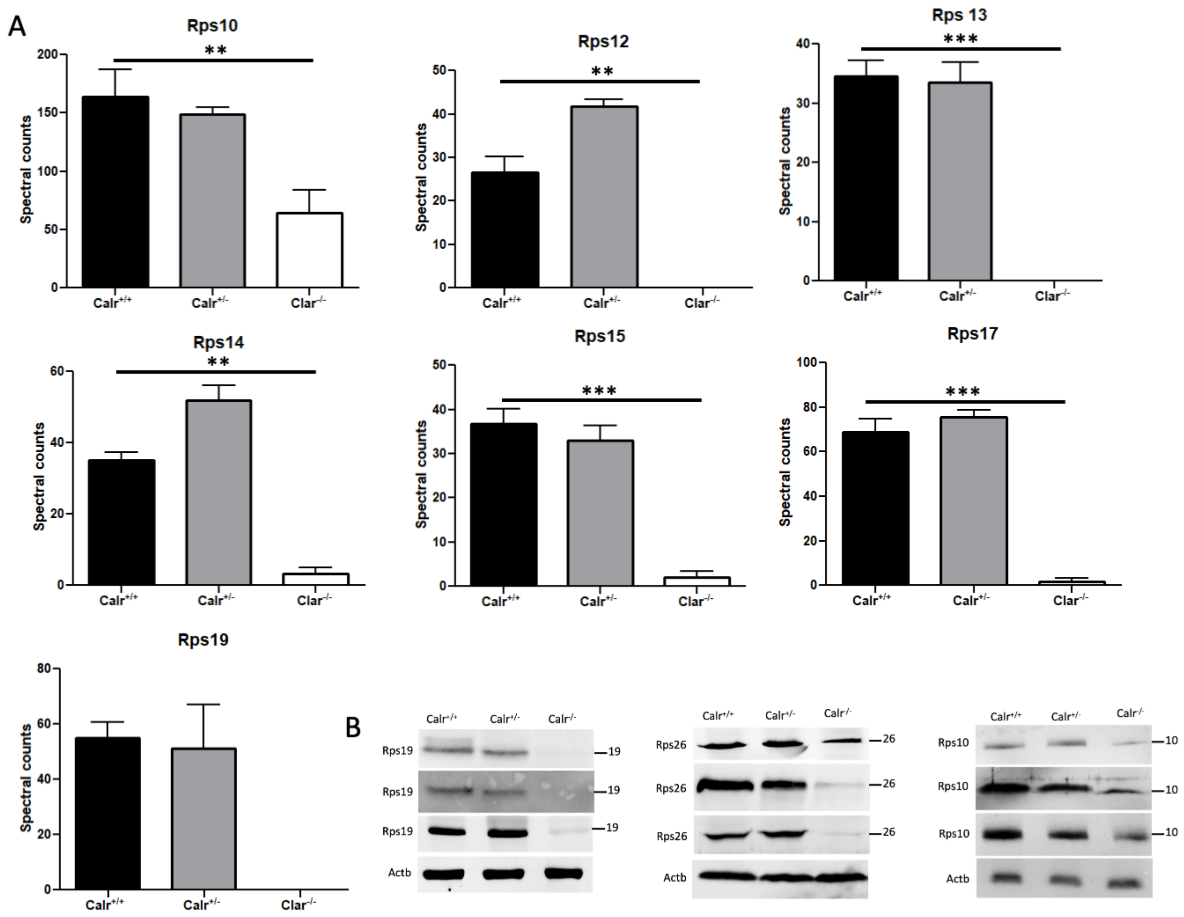

Supplementary Figure S6. (**A**) MS quantification of ribosomal proteins found to be altered in Calr$^{-/-}$ embryonic kidney. The embryos were derived from at least three different pregnant Calr$^{+/-}$ mice. Kidneys from the same genetical background were grouped together. Protein extracts were prepared from embryonic kidneys from the three genotypes (Calr$^{+/+}$, Carl$^{+/-}$, and Calr$^{-/-}$) and subjected to tryptic digest before mass spectrometric analysis. The protein expression quantifications for selected ribosomal proteins are given in the form of bar diagrams. Results are given as the means ± SD of the spectral counts ($p < 0.05$). (**B**) Western blot replicate for the ribosomal protein Rps10, Rps19, and Rps26. Protein extracts from embryonic kidney from the three genotype (Calr$^{+/+}$, Calr$^{+/-}$, Calr$^{-/-}$) were separated on SDS-PAGE and the blots were investigated with the antibodies against the corresponding proteins.
